# Rate thresholds in cell signaling have functional and phenotypic consequences in non-linear time-dependent environments

**DOI:** 10.3389/fcell.2023.1124874

**Published:** 2023-03-21

**Authors:** Alexander Thiemicke, Gregor Neuert

**Affiliations:** ^1^ Department of Molecular Physiology and Biophysics, School of Medicine, Vanderbilt University, Nashville, TN, United States; ^2^ Program in Chemical and Physical Biology, Vanderbilt University, Nashville, TN, United States; ^3^ Department of Biomedical Engineering, School of Engineering, Vanderbilt University, Nashville, TN, United States; ^4^ Department of Pharmacology, School of Medicine, Vanderbilt University, Nashville, TN, United States

**Keywords:** cell signaling, quantitative biology, single cell, time lapse microscopy, dynamic environments, flow cytometry, rate threshold, systems biology

## Abstract

All cells employ signal transduction pathways to respond to physiologically relevant extracellular cytokines, stressors, nutrient levels, hormones, morphogens, and other stimuli that vary in concentration and rate in healthy and diseased states. A central unsolved fundamental question in cell signaling is whether and how cells sense and integrate information conveyed by changes in the rate of extracellular stimuli concentrations, in addition to the absolute difference in concentration. We propose that different environmental changes over time influence cell behavior in addition to different signaling molecules or different genetic backgrounds. However, most current biomedical research focuses on acute environmental changes and does not consider how cells respond to environments that change slowly over time. As an example of such environmental change, we review cell sensitivity to environmental rate changes, including the novel mechanism of rate threshold. A rate threshold is defined as a threshold in the rate of change in the environment in which a rate value below the threshold does not activate signaling and a rate value above the threshold leads to signal activation. We reviewed p38/Hog1 osmotic stress signaling in yeast, chemotaxis and stress response in bacteria, cyclic adenosine monophosphate signaling in Amoebae, growth factors signaling in mammalian cells, morphogen dynamics during development, temporal dynamics of glucose and insulin signaling, and spatio-temproral stressors in the kidney. These reviewed examples from the literature indicate that rate thresholds are widespread and an underappreciated fundamental property of cell signaling. Finally, by studying cells in non-linear environments, we outline future directions to understand cell physiology better in normal and pathophysiological conditions.

## 1 Introduction

**FIGURE 1:**
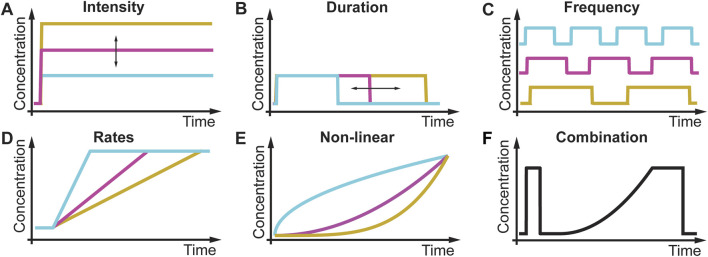
Different environmental dynamics in biology and laboratory research. **(A)** In a dose-response experiment, the intensity of a stimulus changes acutely. **(B)** Duration of stimulus changes. **(C)** Frequency of stimulus changes. **(D)** Rate of the stimulus changes. **(E)** Non-linearity of the stimulus changes. **(F)** Each of these stimulus patterns can be combined to generate infinite numbers of environmental dynamics.

**FIGURE 2:**
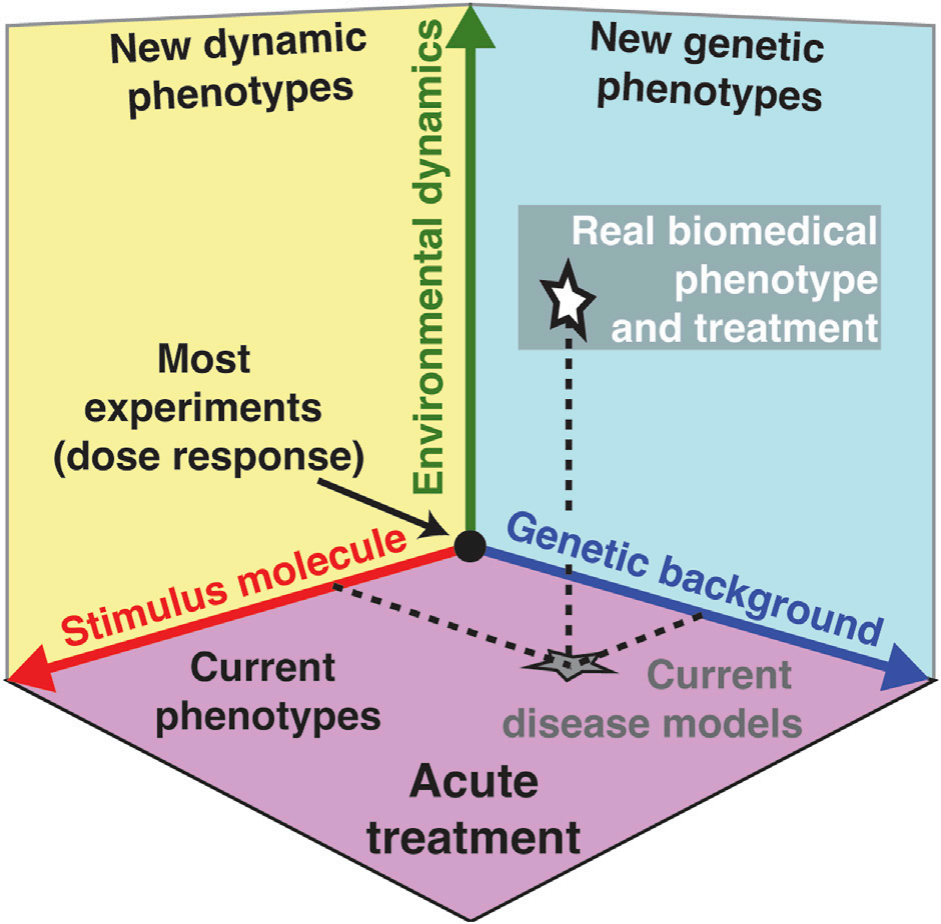
Environmental dynamic of the stimulus molecule as a new dimension in biomedical research. Phenotype relationship between stimulus molecules or drugs (red axis), genetic background (blue axis), and environmental dynamics (green axis). Each combination of these three axis can define a unique phenotype. Because current environmental dynamics often only consider dose-response experiments using acute environmental changes, the green axis effectively projects onto the purple plane that describes most observed present phenotypes, disease models, and treatment regimes (gray star). Environmental dynamics add variety to the types of environmental changes investigated (green arrow) and increase the dimensionality of biological knowledge to new dynamic phenotypes (yellow plane) and new genetic phenotypes (cyan plane), thereby providing a more accurate picture of physiologically relevant biology and biomedicine (white star).

All living cells sense and respond to environmental changes (Lim et al., 2014; Alberts et al., 2015; Murugan et al., 2021). Concentrations of physiologically relevant external stimuli such as nutrients, chemoattractants, cytokines, hormones, growth factors, morphogens, and environmental stressors (Table 1) change in diverse patterns that include variations in the intensity, duration, time between sequential stimulations, rate of change, non-linearity, and combinations of different dynamics (Figure 1; Table 2). As a result, cell responses can change as a function of different gradual and acute environments. Despite these facts, most ongoing biological research measures responses to a limited number of instantaneously changing environments (dose-response curve) in cell populations (Figure 1A) (Lim et al., 2014; Alberts et al., 2015). These studies often use normal or mutant cells to understand how genetic differences or disease impact signaling response and cell phenotype. In Figure 2, we conceptually illustrate the phenotype relationship between stimulus molecules (red axis), genetic backgrounds (blue axis), and dynamic change of the stimulus molecule (green axis). The stimulus molecules axis represents any molecule or molecule combinations that can activate or repress a process in a cell, causing a phenotypic change. This axis includes all drugs and their combination that can impact the cell. This number is infinite, given the various molecules and drugs. The genetic background axis represents any genetic change affecting cell behavior and phenotype. It is also easy to assume that unique genetic constellations are infinite. The third axis presents the different dynamic changes of the stimulus molecule with which any given stimulus molecule can change over time for any given genetic background (Figure 1B–F). However, most biomedical laboratory research uses a finite set of acute changes in the stimulus molecule concentrations (Figure 1A). A typical example of such a study is the classical dose-response experiment that may use a dozen different concentrations (Figure 2, black circle).

**TABLE 1:** Environment type that changes over time.

Environment type	Citations
Nutrients	Block et al. (1983), Segall et al. (1986), Tu et al. (2008), Shimizu et al. (2010)
Chemoattractants	Wang et al. (2012), Chang and Levchenko (2013), Sgro et al. (2015)
Cytokines	Luecke et al. (2021), Meizlish et al. (2021)
Hormones	Bratusch-Marrain et al. (1986), Polonsky et al. (1988), Fernandez and Torres-Alemán (2012), Kubota et al. (2012), Kubota et al. (2018), Noguchi et al. (2013), Sano et al. (2016)
Growth factors	Sasagawa et al. (2005), Fujita et al. (2010), Ji et al. (2010), Avraham and Yarden (2011), Fernandez and Torres-Alemán (2012), Sorre et al. (2014)
Morphogens	Wang et al. (2012), Sorre et al. (2014), Dubrulle et al. (2015), Sagner and Briscoe (2017), Tewary et al. (2017); Hill (2018), Heemskerk et al. (2019), Li and Elowitz (2019), Mateus et al. (2020), Hashmi et al. (2022)
Environmental stressors	Neuhofer and Beck (2005), Muzzey et al. (2009), Pelet et al. (2011), Young et al. (2013), Carlström et al. (2015), Nguyen-Huu et al. (2015), Goulev et al. (2017), Granados et al. (2017), Firsov and Bonny (2018), Thiemicke et al. (2019), Jashnsaz et al. (2020), Jashnsaz et al. (2021), Johnson et al. (2021), Thiemicke and Neuert (2021)

**TABLE 2:** Different dynamics of the same environment.

Environment dynamics	Citation
Intensity	Hersen et al. (2008), Mettetal et al. (2008), Macia et al. (2009), Muzzey et al. (2009), Pelet et al. (2011), Neuert et al. (2013), Granados et al. (2017), Jashnsaz et al. (2020)
Rate of change	Block et al. (1983), Sasagawa et al. (2005), Muzzey et al. (2009), Fujita et al. (2010), Shimizu et al. (2010), Pelet et al. (2011), Kubota et al. (2012), Kubota et al. (2018), Wang et al. (2012), Young et al. (2013), Sorre et al. (2014), Sgro et al. (2015), Heemskerk et al. (2019), Thiemicke et al. (2019), Johnson et al. (2021), Thiemicke and Neuert (2021)
Duration	Hersen et al. (2008), Mettetal et al. (2008), Mitchell et al. (2015), Gunne-Braden et al. (2020)
The time between sequential stimulations	Hersen et al. (2008), Mettetal et al. (2008), Mitchell et al. (2015), Rahi et al. (2017), Jashnsaz et al. (2020), Johnson et al. (2021)
Combinations of environment dynamics	Bandara et al. (2009), Wang et al. (2012), Sgro et al. (2015), Jashnsaz et al. (2020), Jashnsaz et al. (2021), Johnson et al. (2021)

In contrast, cells of any organism or in any natural environment experience a wide variety of gradual changes in external stimuli. Current treatments and therapies are based on observed phenotypes (purple plane) and current disease models (grey star) in such acute treatments, potentially missing or misinterpreting the response of a cell to the stimulus under physiological conditions. For example, if one uses a dozen concentrations from the previously mentioned dose-response experiment and alters the concentration acutely every 1 min for 30 min, then there are 12^30^ = 2.3 * 10^32^ unique combinations possible. This simple example demonstrates the possible infinite space of modulating the environment of cells in a non-acute manner. This new dimension of dynamic changes of the stimulus molecule may dramatically expand our phenotypic space currently not explored in the laboratory (white star). Including this critical dimension will allow us to observe physiologically relevant phenotypes in different dynamic phenotypes (yellow plane) and under different genetic backgrounds (cyan plane). By designing experiments that consider this new dimension of dynamic changes of the stimulus molecule, we expect to better characterize the accurate and relevant biomedical phenotype (white star) compared to current disease models (grey star) that do not fully consider this new dynamic dimension. As an example of the latest insights gained from dynamic temporal environments, we will focus on the newly discovered concept of a rate threshold. Similar to a concentration threshold, where a concentration above a certain value activates signaling, a rate threshold is a value in the rate of change in the environment that needs to be crossed to activate signaling. We argue that a rate threshold is a novel and phenotypically important mechanism in cell signaling that is relevant in gradual and non-acute changing environments. In this review, we will focus on pathways that respond to gradual environmental changes and describe different examples from the literature to illustrate the importance of non-acute and gradually changing environments from bacteria to humans. We will start with a yeast Mitogen-Activated Protein Kinase (MAPK) pathways rate sensitivity and rate threshold as a paradigm for all the remaining pathways.

## 2 Concentration and rate thresholds regulate yeast stress response

**FIGURE 3:**
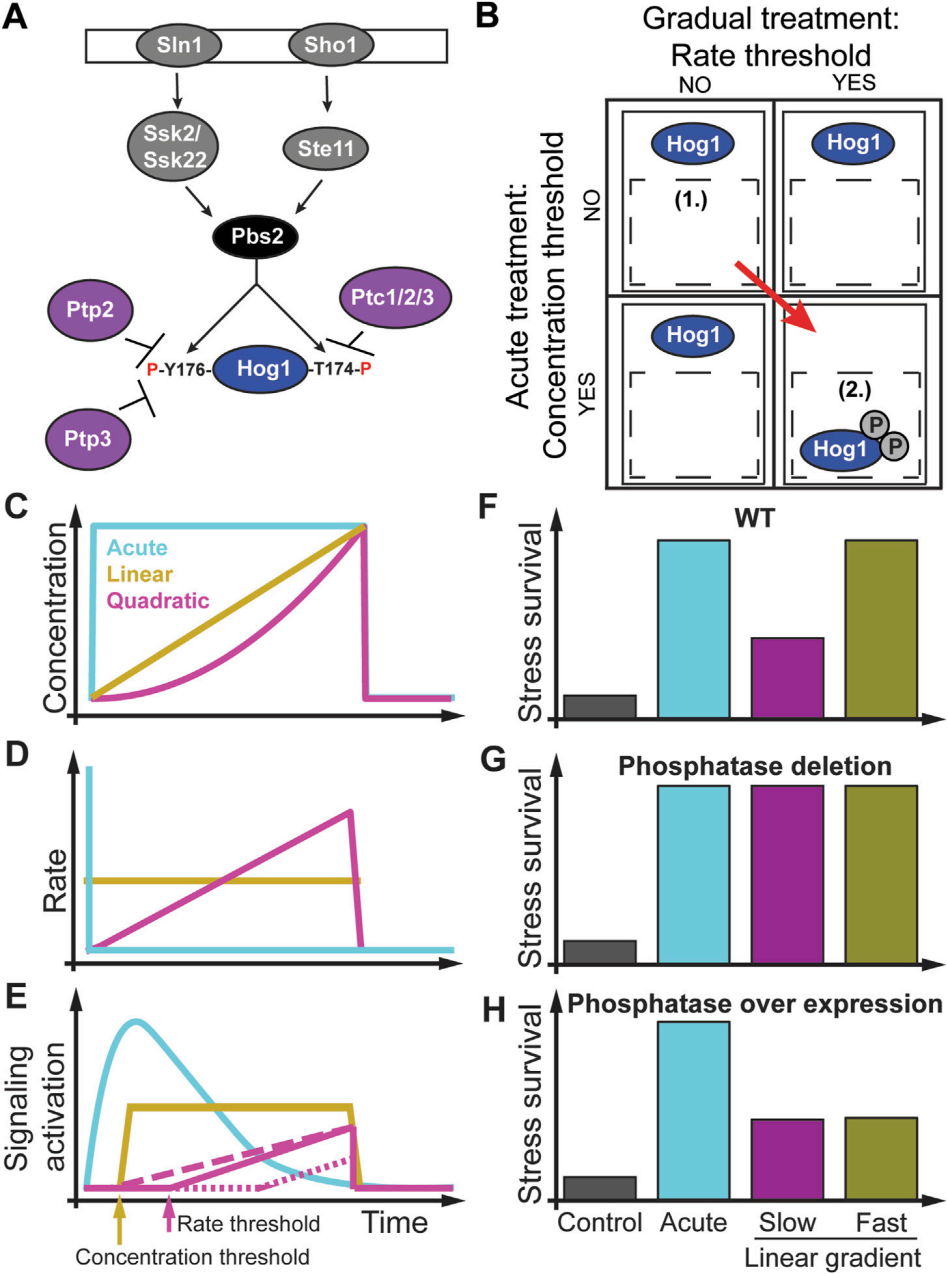
Rate threshold encoding in the yeast HOG pathway. **(A)** Components of the High Osmolarity Glycerol MAPK pathway. **(B)** Non-acute treatments such as linear and quadratic osmolyte gradients discovered a rate threshold with a concentration threshold regulating Hog1 MAPK through an AND logic. **(C)** Different osmolyte concentration profiles as a function of time. **(D)** Rate changes for these concentration changes. **(E)** Hog1 signaling activation depends on the cell environmental dynamics and pathway mutants. For a linear concentration increase with a rate above the rate threshold (sand line), signal activation increases after a concentration threshold is met (sand arrow). For a quadratic concentration increase (magenta line) resulting in a linear increase in the rate, the concentration threshold is met earlier than the rate threshold (magenta arrow), and Hog1 gets activated (magenta solid line). Phosphatase deletion removed the rate threshold (magenta dashed line). Overexpression of the phosphatase increases the rate threshold (magenta dotted line). **(F)** A stress-resistant assay shows increase survival of acute (cyan bar) and fast (yellow bar) but not slow (magenta bar) stressed cells. **(G)** Deletion of phosphatase makes cells resistant to rate changes. **(H)** Overexpression of phosphatase makes cells hyper-sensitive to rate changes but not to acute changes. Adapted from (Johnson et al., 2021).

The high osmolarity glycerol (HOG) MAPK pathway in the budding yeast *Saccharomyces cerevisiae* (Figure 3A) is an ideal model system (Brewster et al., 1993; Saito and Posas, 2012; Brewster and Gustin, 2014) for addressing the question how do rate sensitivity and rate thresholds impact cell signaling and phenotype (Thiemicke et al., 2019; Jashnsaz et al., 2020; Johnson et al., 2021). At the molecular level, pioneering studies found that one signaling branch is activated through the Synthetic Lethal of N-end rule (Sln1) osmosensing histidine protein kinase leading to activation of the MAPKKK’s Suppressor of Sensor Kinase (Ssk2) and its paralog Ssk22 and converging on the MAPKK Polymyxin B Sensitivity (Pbs2) kinase through several intermediate proteins. Another signaling branch is activated through the Synthetic, High Osmolarity-sensitive (Sho1) signaling protein osmosensor that activates the STErile Signal transducing MEK kinase (STE11) converging on Pbs2 (Maeda et al., 1994; Posas et al., 1996; Posas and Saito, 1997; Macia et al., 2009; Saito and Posas, 2012). Activated Pbs2 then dually phosphorylates the evolutionarily conserved MAPK Hog1 on threonine residue 174 (T174) and tyrosine residue 176 (Y176) (Saito and Posas, 2012). This dual phosphorylation is required for Hog1 nuclear import (Figures 3A, B) (Ferrigno et al., 1998; Westfall and Thorner, 2006). Once in the nucleus, the activated Hog1 regulates the expression of several hundred stress response genes (Gasch et al., 2000; Rep et al., 2000; Capaldi et al., 2008; Pelet et al., 2011; Neuert et al., 2013; Nadal-Ribelles et al., 2014). Hog1 can be inactivated by the phosphotyrosine-specific phosphatases Ptp2 and Ptp3 and the type 2C protein phosphatases (PP2C) Ptc1, Ptc2, and Ptc3 (Jacoby et al., 1997; Wurgler-Murphy et al., 1997; Mattison and Ota, 2000; Warmka et al., 2001; Young et al., 2002; Mapes and Ota, 2004; Saito and Posas, 2012). Among these phosphatases, only Ptc1 uses an adaptor protein (the Nap binding protein Nbp2) to transiently interact with Pbs2 (Mapes and Ota, 2004; Stanger et al., 2012). The multiple and presumably redundant MAPK phosphatases dephosphorylate and inactivate Hog1, which, along with the termination of upstream signaling after adaptation, results in its return to the cytosol. This knowledge of the Hog1 pathway was established through acute osmotic stress concentration increases that induce Hog1 phosphorylation, activation, and translocation to the nucleus (Figures 3A, B) (Brewster et al., 1993; Ferrigno et al., 1998; Reiser et al., 1999; Hersen et al., 2008; Macia et al., 2009; Muzzey et al., 2009; Pelet et al., 2011; Saito and Posas, 2012; English et al., 2015; Mitchell et al., 2015; Granados et al., 2017). Activated Hog1 controls the regulation of cellular osmoadaptation and survival (Saito and Posas, 2012; Mitchell et al., 2015; Johnson et al., 2021). Because of this behavior of Hog1 nuclear enrichment, single-cell time-lapse microscopy and analysis have proven an excellent and sensitive way to monitor signaling responses to dynamic stimulation patterns in real-time (Figure 3) (Hersen et al., 2008; Mettetal et al., 2008; Muzzey et al., 2009; Patterson et al., 2010; Munsky et al., 2012; Mitchell et al., 2015; Granados et al., 2017; Thiemicke et al., 2019; Jashnsaz et al., 2021; Johnson et al., 2021). This single-cell analysis of Hog1 nuclear localization response to instant osmotic stress resulted in the discovery of perfect adaptation in this pathway (Figures 3C–E) (Hersen et al., 2008; Mettetal et al., 2008; Muzzey et al., 2009). Perfect adaptation means that the pre and post-stimulus signal is identical. Because of adaptation, the Hog1 pathway is sensitive to the concentration and rate of external stimulus. Pioneering studies then used increasing linear gradients of osmolytes to test the adaptation model (Figures 3C–E) (Muzzey et al., 2009). The proposed network motif to achieve perfect adaptation in this pathway is the integral feedback loop (Stelling et al., 2004) by which the cell integrates up internal glycerol and computes the pressure difference between outside and inside the cell (Mettetal et al., 2008; Muzzey et al., 2009). Almost a decade later, several osmolyte rates were used to dissect the contribution of the different upstream Hog1 signaling branches showing how each branch has a slightly different rate dependence in regulating Hog1 nuclear enrichment (Granados et al., 2017). However, neither of these studies considered how these different osmolyte gradients regulate growth or survival phenotypes. Another study using square waves of osmolytes of different duration showed that fast and slow fluctuating osmolyte environments do not alter yeast doubling time. However, fluctuations with intermediate durations resulted in a fourfold reduction in yeast growth rate, showing that environmental fluctuations have phenotypic consequences (Mitchell et al., 2015). Several labs developed novel cell culture assays to expose cells to non-acute environmental changes, followed by quantitative experiments to investigate molecular mechanisms in non-acute environmental conditions (Figures 3C, D) (Block et al., 1983; Muzzey et al., 2009; Fujita et al., 2010; Shimizu et al., 2010; Pelet et al., 2011; Kubota et al., 2012; 2018; Wang et al., 2012; Noguchi et al., 2013; Sgro et al., 2015; Mokashi et al., 2019; Thiemicke et al., 2019; Krause et al., 2021). The novel experimental setup developed by Thiemicke et al. recently investigated how Hog1 signaling and cell viability are impacted by different rates of osmolyte gradients (Figures 3C–H) (Johnson et al., 2021). In Figure 3C, the authors applied acute (cyan line), linear (sand line), and quadratic (magenta line) concentration changes with the same final concentration and duration to yeast cells. These concentration changes result in no rate changes for acute stresses (cyan line), a constant rate change for a linear concentration increase (sand line), or a linear rate increase for a quadratic concentration increase (magenta line) (Figure 3D). As a result of these concentration profiles (Figure 3E), Hog1 signal activation adapted perfectly upon acute concentration changes (cyan line). Upon a linear stress increase, Hog1 activates with a delay after reaching a concentration threshold (sand arrow) followed by constant signal amplitude (sand line). Interestingly, exposing yeast cells to a quadratic stress increase resulted in a longer delay in Hog1 activation due to a threshold in the rate (magenta line and arrow). Only after a threshold rate was reached did the Hog1 signaling increase linearly. In detailed experiments, they showed that Hog1 nuclear localization depends on an osmolyte concentration and a rate threshold. Additional experiments showed that the Hog1 pathway uses an AND logic to integrate the previously determined concentration threshold (Macia et al., 2009) and the rate threshold. Both thresholds are required to activate Hog1 nuclear localization. Figure 3B shows how different stress treatment dynamics can probe one or the other threshold. The authors then used different linear osmolyte gradients to the same final and total osmolyte concentration below and above the threshold rate, followed by a second severe stress. Using a colony formation assay, they found that cells treated with a pulse or a linear osmolyte gradient above the threshold rate survive ten times better than untreated cells (Figure 3F). However, cells treated with a linear increasing osmolyte gradient below the rate threshold survived only five times better than untreated cells, demonstrating that the rate of the gradient determines cell survival. In these experiments, the dose defined as the integrated NaCl exposure is identical between the different experiments, but the viability phenotype is different. They then investigated the molecular basis of this rate threshold by performing a targeted genetic knockout screen and identified the phosphatase Ptp2 but not the seemingly redundant Ptp3 as a rate threshold regulator leading to earlier activation of Hog1 nuclear localization only dependent on the threshold concentration (Figure 3E, dashed magenta line). The knockout of Ptp2 was then rescued by varying the expression level of Ptp2, showing that the threshold rate is proportional to the expression level of Ptp2 (dotted magenta line). Subsequent colony formation experiments showed that the deletion of Ptp2 made cells resistant to osmolyte gradients (Figure 3G). In contrast, overexpression of Ptp2 made cells hypersensitive to osmolyte gradients but not to acute osmolyte changes (Figure 3H). These results demonstrate that gradient environmental changes regulate cellular phenotypes. Yeast cells use a novel rate threshold mechanism to differentiate between different rates of stimulus increase. Moreover, “redundant phosphatases” have specific non-redundant functions only detectable in non-acute environmental conditions. We speculate that a possible network motives that could give rise to such a rate threshold in an adapting system is a combination of a Hill function with a negative feedback loop or with an incoherent feed forward loop (Milo et al., 2002; Ma et al., 2009; Rahi et al., 2017). In the context of signal transduction, a Hill function describes the relationship between the concentration of a ligand and the non-linear activation of a downstream signaling protein. A negative feedback loop is the regulation of an upstream signaling protein through a downstream signaling protein. An incoherent feed forward loop has the property to activate both a target signaling protein and an inhibitor of that proteins, which then inhibits the target signaling protein. This study hypothesized that the novel rate threshold mechanism in cell signaling might be prevalent in other pathways and organisms, which is the focus of this review article. We next describe how rate sensitivity and thresholds are prevalent in bacterial chemotaxis.

## 3 *E. coli* chemotaxis nutrient sensing

**FIGURE 4:**
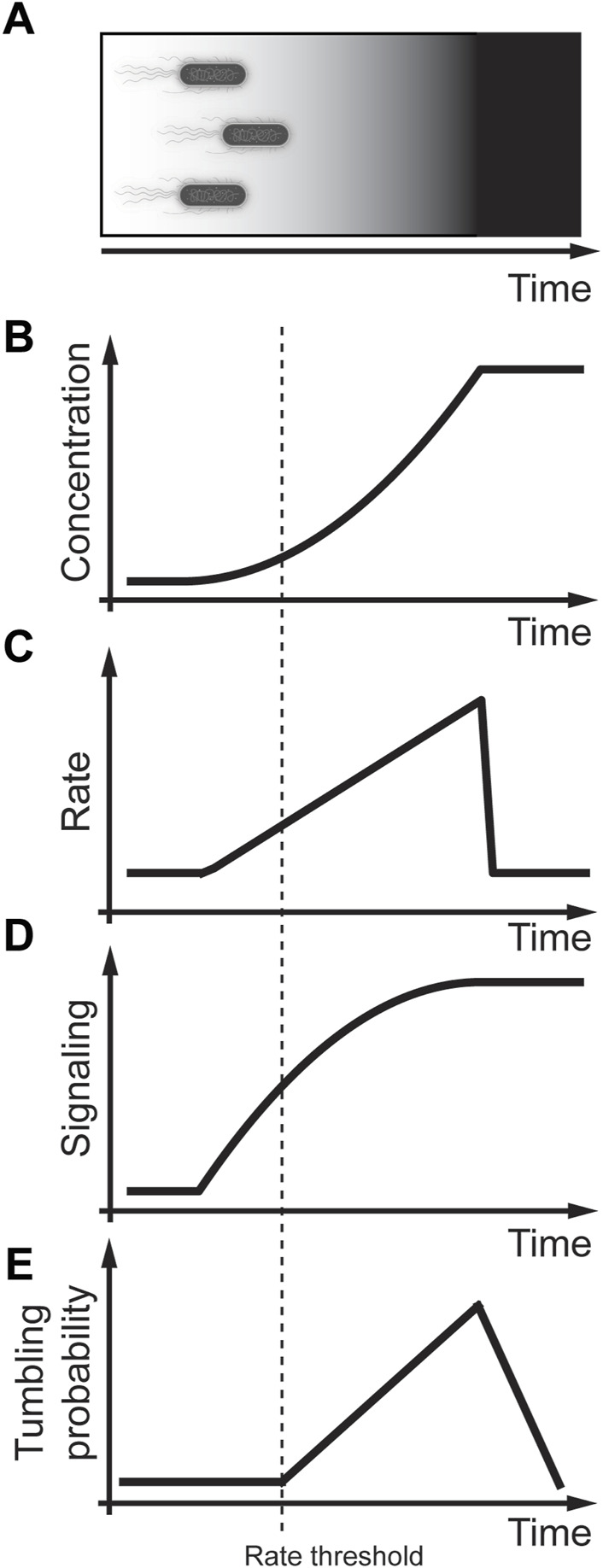
Rate threshold encoding in *E. coli* chemotaxis. **(A)** Physiological context in which *E. coli* swim towards a food source that increases non-linearly. **(B)** Modeling non-linear increase in nutrient concentration in a cell culture experiment. **(C)** Linear increase in the rate of nutrient concentration. **(D)** Increase in the rate result in signaling activation. **(E)** Bacterial tumbling probability only activates after detecting a threshold rate of nutrients. Adapted from (Block et al., 1983; Shimizu et al., 2010).

Chemotaxis is a process that allows bacteria to sense nutrients in their surrounding by randomly sampling their environments and then measuring the nutrient concentration over time (Figure 4A) (Adler and Alon, 2018; Tu and Rappel, 2018). Although chemotaxis in bacteria is molecularly well understood (Waite et al., 2018; Karmakar, 2021), the physiological relevance leaves many open questions (Colin et al., 2021; Keegstra et al., 2022). The core signal transduction pathway responsive to chemotaxis in *E. coli* is reviewed in detail by (Waite et al., 2018; Karmakar, 2021). Here we briefly summarize the chemotaxis pathway that consists of a membrane-associated receptor kinase complex A that can sense an external ligand concentration [L]. Activation of complex A regulates the autophosphorylation activity resulting in a phosphate transfer to the response regulator CheY leading to phosphorylated ChY (ChY-P). ChY-P then interacts with the flagellar motor M to control swimming. The activity of CheY is determined by the phosphorylation of CheY through the histidine kinase ChrA and dephosphorylation through the phosphatase CheZ. The receptor kinase complex A is regulated through feedback consisting of the methyltransferase CheR and the methylesterase CheB that modified the number of methylated glutamyl residues. Early studies of quantifying *E. coli* tumbling frequency discovered that acute exposure to nutrients resulted in an instant response that perfectly adapted to the initial conditions within a few seconds (Block et al., 1982). This response was explained through a model by an integral feedback loop from the receptor kinase complex A activating the phosphorylated methylesterase/deamidase CheB-P. ChB, ChB-P, and methyltransferase CheR then provide feedback to regulate the receptor methylation of the receptor kinase complex A (Shimizu et al., 2010). A vital property of an adaptive system is that it is sensitive to the concentration and the rate of change of the external ligand.

Further understanding of this adaptive behavior was acquired through carefully controlled *in vitro* experiments using exponentially increasing and decreasing gradient profiles of external ligands (Figures 4B, C) (Block et al., 1983; Segall et al., 1986; Shimizu et al., 2010). Shimizu et al. (2010) performed elegant fluorescence resonance energy transfer (FRET) experiments between CheY, and CheZ, genetically tagged with a yellow fluorescent protein (YFP) and cyan fluorescent protein (CFP), respectively. The measured FRET signal is a live cell readout that approximates CheY-P, the phosphorylated form of CheY. These studies showed that even small increases in the rate of the external ligand lead to CheY signaling (Figure 4D) (Shimizu et al., 2010). In contrast, carefully controlled experiments measuring the rotational probability of the flagellum by Block et al. (1983) showed that there is a potential rate threshold that the ligand needs to overcome to activate the flagellum motor (Figure 4E). This rate threshold was explained as a time delay required to measure the rate changes (Block et al., 1983). An alternative explanation is that the amount of ChY-P or rate change of ChY-P might need to overcome a threshold similar to the rate threshold in MAPK signaling in yeast cells (Johnson et al., 2021). To address this hypothesis, future studies need to expose *E. coli* to different concentration gradients and measure ChY-P as a signaling readout and the *E. coli* tumbling frequency as an activity readout. Ideally, these experiments could then be repeated in other mutant strains of the chemotaxis pathway to identify potential proteins that regulate the rate threshold.

## 4 Bacterial *B. subtilis* stress response

**FIGURE 5:**
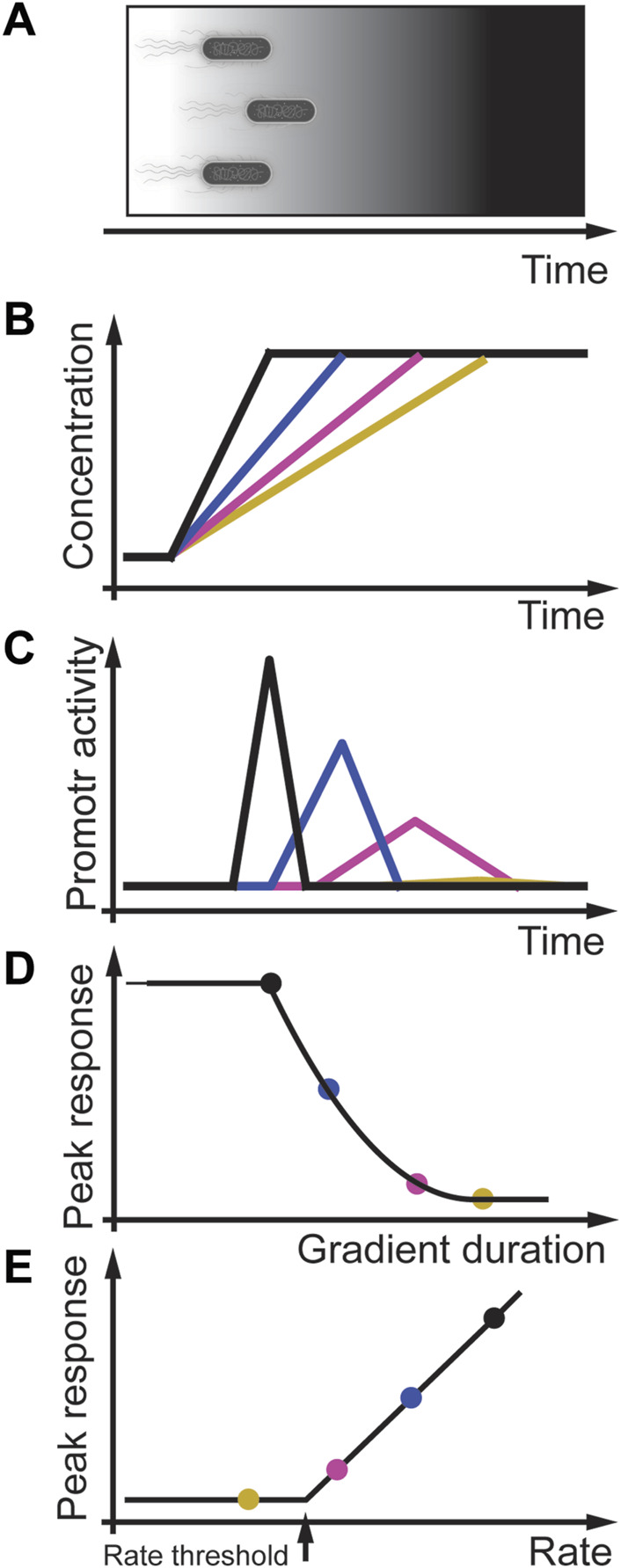
Rate threshold encoding in *B. subtilis* stress response. **(A)** Physiological context in which *B. subtilis* experience an increasing stress gradient. **(B)** *In-vitro* modeling of different linear gradients of osmotic stress. **(C)** Gene expression promotor activity is transient and dependent on the stress rate. **(D)** Peak response (colored solid circles) decreases with gradient duration and decreasing rate. **(E)** Peak response as a function of the rate indicate that *B. subtilis* stress response may encode a rate threshold. Adapted from (Young et al., 2013).

Another example of rate sensitivity in prokaryotes is the general stress response in the bacterium *B. subtilis* (Figure 5A) (Hecker et al., 2007). In this bacterium, different stressors activate the stressosome that regulates the availability of the serine/threonine-protein kinase RsbT. RsbT phosphorylates the phosphoserine phosphatase RsbU, which then dephosphorylates RsbV, an anti-sigma-B factor antagonist. The binding of RsbV to the serine-protein kinase RsbW results in the release of sigma factor σ^B^ that activates target promotors, including its operon, the phosphatase RsbX, and induces other genes of the environmental stress response. Expression of RsbX negatively regulates and feedbacks to RsbT. This feedback is essential for the adaptive behavior of *B. subtilis* to instant environmental stress (Young et al., 2013). In carefully controlled experiments, Young et al. (2013) showed that the amplitude of a transcriptional single-cell reporter is proportional to the intensity of the acute and instant stressors NaCl, ethanol, and butanol (Figures 5B, C) consistent with population studies (Boylan et al., 1993). As expected from an adaptive system, when NaCl or ethanol stress increases at different rates, the amplitude of the σ^B^ promotor response changes proportional to the gradient rate (Figure 5D) (Young et al., 2013). However, at very shallow rates to a high-stress concentration of NaCl or ethanol, the promotor response was similar to non-stressed cells, indicating that stress response in *B. subtilis* may also be regulated by a rate threshold (Figure 5E). Further studies must be performed using different linear and quadratic stress gradients in normal and mutated bacteria to investigate this potential rate threshold mechanism.

## 5 Rate sensitivity in amoebae response

**FIGURE 6:**
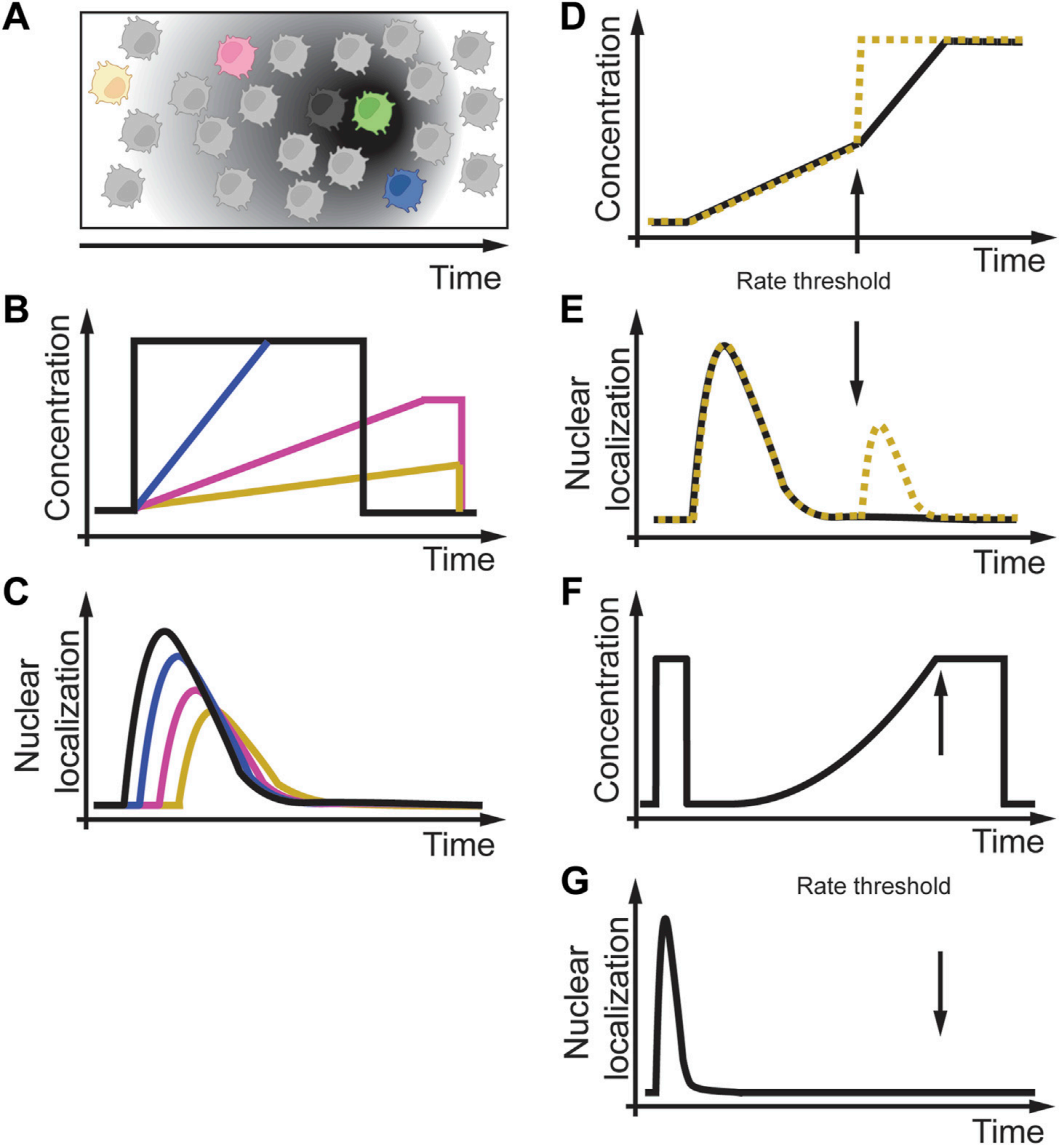
Rate sensitivity and rate threshold in amoeba *Dictyostelium discoideum*. **(A)** A *D. discoideum* cell (green) is secreting cAMP over time, and cells at different distances (black, blue, magenta, yellow cells) experience different cAMP gradients over time. **(B)** *In-vitro* modeling of different acute and linear cAMP gradients over time. **(C)** Different temporal cAMP gradients result in delayed and reduced activity of cAMP signaling. **(D)** Exposure of *Dictyostelium* cells to two different cAMP profiles with the same initial rate and a subsequent faster rate (black solid line) or an acute increase (yellow dashed line). **(E)** *Dictyostelium* cells respond to a slow first treatment rate but only to an acute second treatment (yellow dashed line) and not to a slow second treatment (black solid line), indicating a rate threshold. **(F,G)** Non-linear gradients below a possible threshold rate do not activate cAMP signaling. Adapted from (Wang et al., 2012; Sgro et al., 2015).

Besides single-cell organisms, rate sensitivity, and rate thresholds were also observed in cells of multicellular organisms. This section will focus on the chemoattractant cyclic adenosine monophosphate (cAMP) sensitivity of single *Dictyostelium* cells (Figure 6A) (Artemenko et al., 2014; Nichols et al., 2015). These single cells can use cell-to-cell communication through cAMP to coordinate collective cell behavior, the basis for the slug and fruit body formation process. Figure 6A illustrate a situation in which a cell secretes cAMP (green cell), and cells in the neighborhood (black, blue, magenta, yellow cells) sense different temporal gradient in cAMP dependent on their distance to the secreting cell (grey gradient). The signaling process involves cAMP binding to cAMP-specific heterotrimeric guanosine triphosphate–binding protein (G protein)–coupled receptors (GPCRs). These GPCRs then transiently activate phosphoinositide 3-kinase (PI3K), which then adapts to persistent and constant cAMP concentrations. A fundamental question is how individual *Dictyostelium* cells sense cAMP changes in their environment over time, as in the process from a single cell to a multicellular organism. To address this question in a controlled environment, Wang et al. (2012) and Sgro et al. (2015) designed experiments in which they changed the dynamics of the cAMP concentrations over time (Figures 6B, D, F). Wang et al. (2012) developed a sophisticated microfluidic chip to generate acute and slowly changing cAMP concentrations at different rates (Figure 6B). They used a phosphatidylinositol 3,4,5-trisphosphate (PIP3)–specific biosensor to monitor its kinetics to the plasma membrane as a live cell readout of cAMP signaling (Figure 6C). Similar to previous studies, they observed rapid transient and adapting biosensor translocation. With this experimental setup, they experimentally and computationally studied the cAMP signaling response to better understand the underlying effective signaling network structure. As part of their studies, they used linearly increasing cAMP gradients of different rates. They experimentally found that decreasing the cAMP rate led to a delay and reduced amplitude in biosensor readout (Figure 6C). Interestingly, when they used two different subsequent rates (Figure 6D), of which the first rate was fast. Still, the second rate was slow (Figure 6D, black line), the cell did not respond to the second slow rate cAMP stimulus (Figure 6E, black line), whereas if the second rate was fast (Figure 6D, yellow dashed line), cells responded (Figure 6E, yellow dashed line). Similarly, in *Dictyostelium* cells, Sgro et al. (2015) showed that signaling due to an exponential cAMP increase starts to oscillate above a certain rate threshold (Figures 6F, G). These results indicate that cAMP signaling in *Dictyostelium* cells is rate sensitive and contains a rate threshold. However, the mechanism and protein(s) decoding these rate thresholds within the same or different cells is elusive and requires further studies.

## 6 Temporal dynamics of growth factors in mammalian cells

**FIGURE 7:**
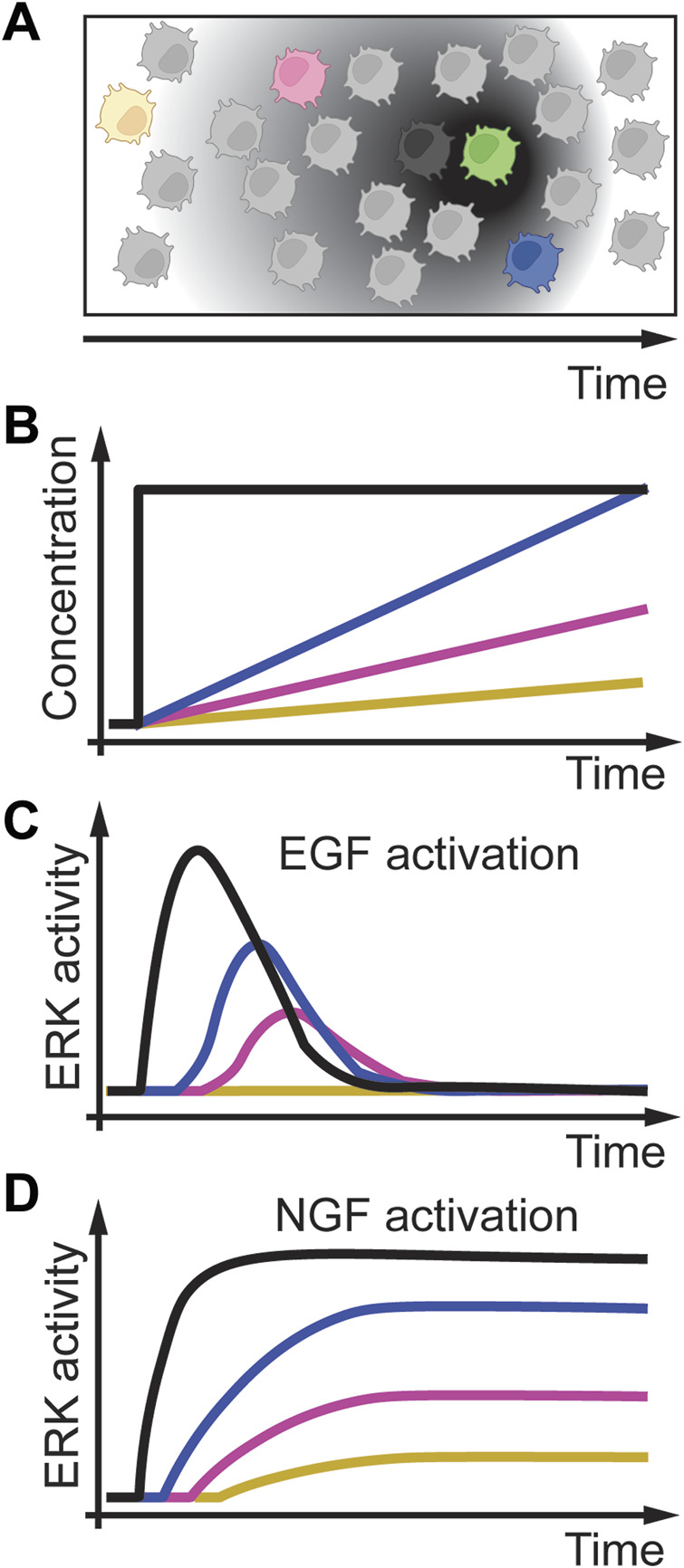
Temporal dynamics of growth factors. **(A)** A cell (green) secreting growth factors such as EGF or NGF over time, and cells at different distances (black, blue, magenta, yellow cells) experience different growth factor gradients over time. **(B)** *In-vitro* modeling of cells exposed to acute and linear gradients of EGF or NGF. **(C)** EGF activation of ERK results in adaptive signaling with delayed and reduced signaling amplitude. A slow rate of EGF (yellow line) does not result in ERK activation indicating a rate threshold. **(D)** NGF activation of ERK results in a reduced speed of activation but no adaptation. Adapted from (Sasagawa et al., 2005; Fujita et al., 2010).

Growth factors are molecules that stimulate cell proliferation and growth. These molecules change over time in various tissues and organisms (Figure 7A) (Fernandez and Torres-Alemán, 2012; Li and Elowitz, 2019; Koseska and Bastiaens, 2020). A long-studied system of growth factor signaling is the dynamic activation of the extracellular-signal-regulated kinase (ERK) signaling network through the epidermal growth factor (EGF) and nerve growth factor (NGF) (Avraham and Yarden, 2011). Extracellular EGF binds to the EGF receptor (EGFR), which then, through several proteins, activates the Rat sarcoma virus (RAS) protein. RAS then transiently activates ERK. When extracellular NGF is present, it can bind to the Tropomyosin receptor kinase A (TrkA). TrkA then activates Ras-related protein 1 (Rap1), resulting in sustained ERK activation. Traditional studies used PC12 cells, a rat pheochromocytoma cell line, to model this pathway response to growth factors. They applied acute concentration changes of EGF (Figure 7B, black line) that resulted in adaptive ERK signaling (Figure 7C, black line). In contrast, acute increasing concentrations of NGF resulted in sustained ERK signaling (Figure 7D, black line). To better understand this pathway and to validate computational predictions, Sasagawa et al. (2005) exposed PC12 cells to linearly increasing concentrations of EGF and NGF at different rates (Figure 7B, colored lines). As expected from an adaptive system, slower rates of EGF resulted in adaptive but reduced ERK signaling intensity (Figure 7C, colored lines), whereas slow rates of NGF resulted in sustained but slower ERK activation (Figure 7D, colored lines). Interestingly, slow-increasing EGF concentration resulted in no ERK activation, whereas acute EGF increases resulted in robust ERK activation. These results on linear EGF gradients activating ERK were confirmed in a subsequent study from the same group, indicating that ERK signaling through EGF might also be rate threshold dependent (Fujita et al., 2010). In this study, Fujita et al. showed that cell proliferation markers pAKT (phosphorylated Protein kinase B) and Ribosomal protein S6 show rate-sensitive signaling. These results indicate that pAKT and S6 might also have a rate threshold.

In an independent study, Ji et al. (2010) exposed cultured rat hippocampal neurons to acute and gradual increases in the brain-derived neurotrophic factor (BDNF). BDNF is a neuropeptide important for synaptic development and plasticity (Park and Poo, 2012; Wang et al., 2022). BDNF binds and activates the TrkB receptor tyrosine kinase, which activates downstream MAPK, phosphatidylinositol-3 kinase (PI3K), and Phospholipase C, gamma 1 (PLC-γ) pathways. One central question by Ji et al. is how different dynamics of BDNF activate signaling and regulate phenotype. They found that acute administration of BDNF to primary neurons resulted in adaptive phosphorylation of TrkB and ERK. In contrast, a logarithmic increase in BDNF resulted in a gradual and sustained activation of the same proteins. A significant conclusion from this study was that BDNF delivery rate might be the primary regulatory mechanism rather than the absolute BDNF concentration. As a mechanism, they showed that the Ras-MAPK complex is transiently activated in acute and gradual conditions. In contrast, the Rap1-MAPK complex is transiently activated in acute situations and sustainably activated in gradual conditions. Ji et al. also showed that PLC-γ1 and Glycogen synthase kinase-3 beta (GSK-3β) behave similarly to ERK, suggesting that the rate sensitivity is encoded upstream of ERK, PLC-γ1, and GSK-3β signaling. Downstream, differences in ERK signaling were mirrored by the phosphorylation dynamics of the cAMP response element-binding protein (CREB) transcription factor. The different dynamics of CREB phosphorylation and activation are essential for long-lasting synaptic effects of BDNF and result in differential gene expression of immediate early genes. These changes then also affected dendritic growth and the morphological specializations of dendrites of young hippocampal neurons where acute BDNF treatment promotes neurite growth, whereas gradual BDNF treatment regulates neurite branching. Ji et al. then quantified dendritic spine growth to demonstrate the importance of acute and gradual BDNF administration in mature neurons. They showed that acute treatment with BDNF resulted in more spines with larger heads, whereas gradual BDNF regulates the length of spines and the outgrowth of filopodia-like protrusions. They also showed in hippocampal slices as an *in vivo* system that acute BDNF enhances basal synaptic transmission and that gradual BDNF exposure facilitated long-term potentiation (LTP). These results show that the dynamics of BDNF differentially regulate cell signaling and phenotypes and indicate that BDNF signaling is rate sensitive, and this pathway may encode a rate threshold. Further studies are required to establish this rate sensitivity, the rate threshold, and the proteins regulating these processes.

## 7 Spatio-temporal dynamics of morphogens

**FIGURE 8:**
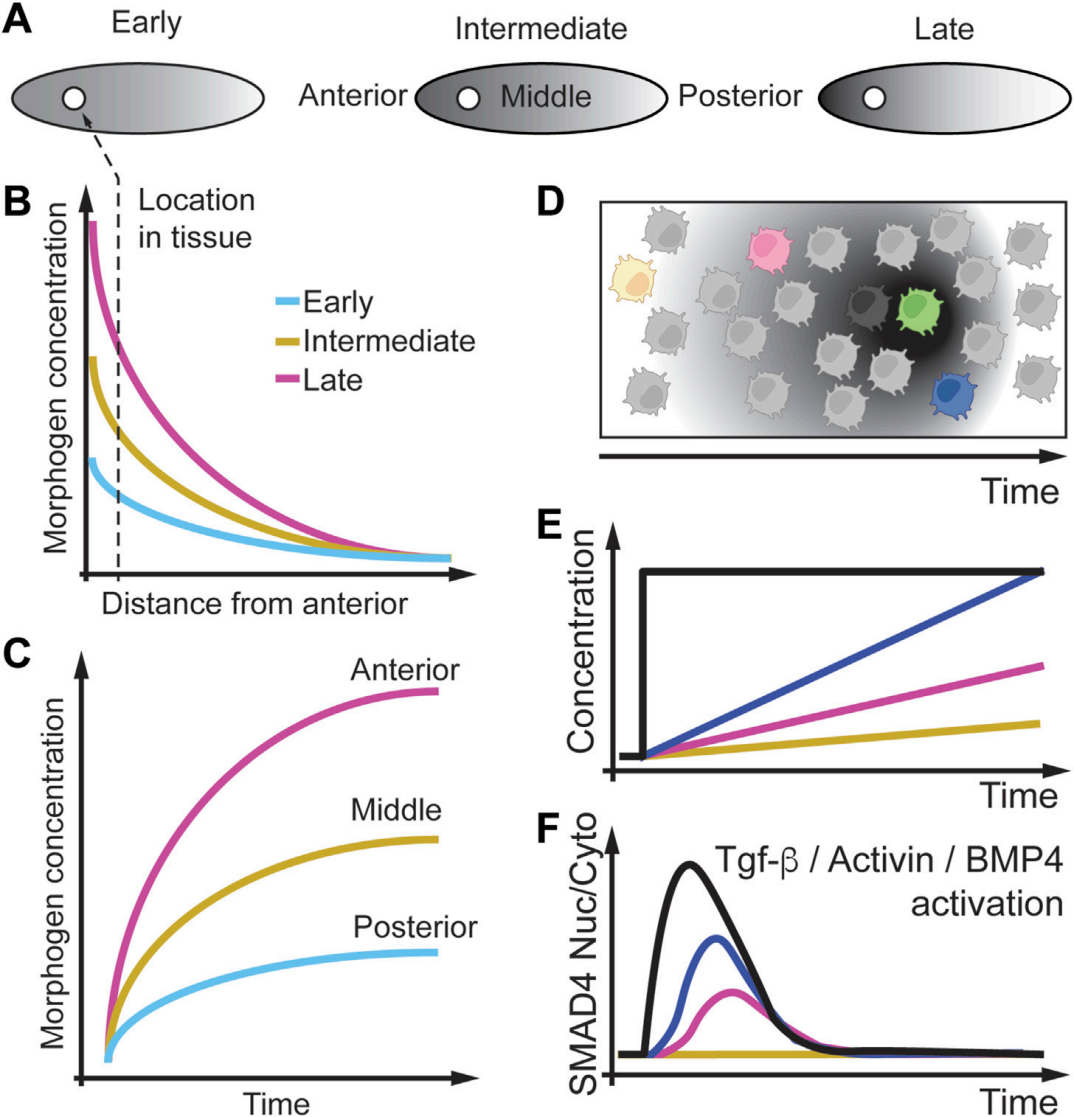
Morphogen gradients change as a function of space and time. **(A)** Morphogen gradients form between the anterior and posterior of a fly embryo and change over time from early to late in development. **(B)** The morphogen gradient decreases along the anterior and posterior axis, starting at a lower concentration at early time points. **(C)** The same data is plotted as a function of time, where nuclei at different positions experience different gradients. **(D)** Modeling morphogen gradients *in vitro*. **(E)** In cell culture different cells can experience different morphogen gradients over time. **(F)** Ratio of nuclear to cytoplasmic SMAD4 as a real-time readout is sensitive to the rate of Tgf-β, Actevin, or BMP administration. Slow rates (yellow line) of Tgf-β, Actevin, or BMP administration does not result in SMAD4 nuclear localization, indicating a rate threshold. Adapted from (Sorre et al., 2014; Heemskerk et al., 2019).

Another important class of signaling molecules that change in space and time are morphogens which are non-uniformly distributed molecules that regulate cell fates during development (Dessaud et al., 2007; Kutejova et al., 2009; Rushlow and Shvartsman, 2012; Li et al., 2013; Dubrulle et al., 2015; Sagner and Briscoe, 2017; Li and Elowitz, 2019; Mateus et al., 2020). In Figure 8A, we depict an example of a developing fly embryo in which a morphogen concentration increases at the anterior pole of the embryo. Over time this increase in concentration results in an increasing concentration gradient from the anterior to the posterior pole of the embryo. Cells (circle) along the embryo will experience morphogen concentration that decreases and become steeper over time (Figure 8B). However, a cell having a fixed position in the embryo will experience an increase in the morphogen concentration over time (Figure 8C). To model how cells respond to changing morphogen gradients over time, cells can be studied *in-vitro,* where the cell environment can be precisely controlled (Figure 8D).

In this review, we highlight some *in vitro* models of embryo development. For example, we choose the transforming growth factor β (TGF-β) signaling pathway as a significant pathway activated by morphogens. Further, we will focus on the ligand proteins TGF-β, bone morphogenetic proteins (BMPs), and Activin and how their gradual concentration profile that changes over time impact signaling and phenotype (Sorre et al., 2014; Heemskerk et al., 2019).

TGF-β signaling is induced by the TGF-β ligands binding to one of the five TGF-β type II receptors. TGF-βs bind the TGF-β type II receptor type 2 (TGFBR2), whereas BMPs bind the BMP receptor type 2 (BMPR2). These receptors are serine/threonine receptor kinases that phosphorylate one of the seven TGF-β type I receptors. A specific TGF-β type I receptor phosphorylates one of the five receptor-regulated Mothers against decapentaplegic homolog (MADHs or SMADs) (R-SMAD’s) (from the small worm *Caenorhabditis elegans* phenotype and MAD family of Mothers Against Decapentaplegic of genes in the fruit fly *Drosophila*). These SMADs can then bind coSMAD SMAD4. R-SMADs (SMAD1, SMAD2, SMAD3, SMAD5, SMAD9) and coSMAD can form complexes in the nucleus and act as transcription factors to regulate target genes. TGF-β signal through SMAD2 and SMAD3, whereas BMPs signal through SMAD1, SMAD5, and SMAD9. Sorre et al. (2014) showed through single-cell time-lapse microscopy experiments that acute TGF-β1 cell stimulation (Figure 8E, black line) leads to rapid Smad4 nuclear localization followed by perfect adaptation and nuclear export (Figure 8F, black line). They then used gradual increases of TGF-β1 (Figure 8E, colored lines) in a staircase administration and observed that Smad4 nuclear localization is rate sensitive (Figure 8F, colored lines). Interestingly, at a low rate of TGF-β1 administration, Smad4 does not localize to the nucleus, which indicates that Smad4 nuclear localization may have a rate threshold.

Another ligand of the TGF-β pathway, BMP, also shows gradual changes over time during mouse and human development (Li et al., 2013). Li et al. (2013) observed qualitatively that BMP in mouse embryos is expressed at different locations with different TGF-β rate increases. Heemskerk et al. (2019) were interested in understanding how different BMP4 and Activin gradients that change over time regulate cell differentiation of human embryonic stem cells (hESCs). They treated these stem cells with BMP4 or Activin acutely or with linearly increasing concentrations (Figure 8E). Heemskerk et al. found that SMAD4 nuclear localization increases rapidly and adapts slowly when cells are treated with BMP4 but adapts quickly when treated with Activin (Figure 8F). However, cells respond slowly when treated with a linear increase of BMP4 or Activin. For Activin, they showed that the rate of BMP4 administration is directly proportional to the rate of SMAD4 nuclear localization. These data indicate that BMP4 and Activin administration rates can modulate SMAD4 signaling and probe a potential rate threshold in SMAD4 signaling. In future studies, one could, for example, expose these cells to different rates of TGF-β1, Activin, and BMP4 to better understand the adaptive SMAD4 behavior, identify a potential rate threshold, and discover the proteins regulating the adaptation and rate threshold mechanisms of SMAD4 signaling.

## 8 Temporal dynamics of glucose and insulin signaling

**FIGURE 9:**
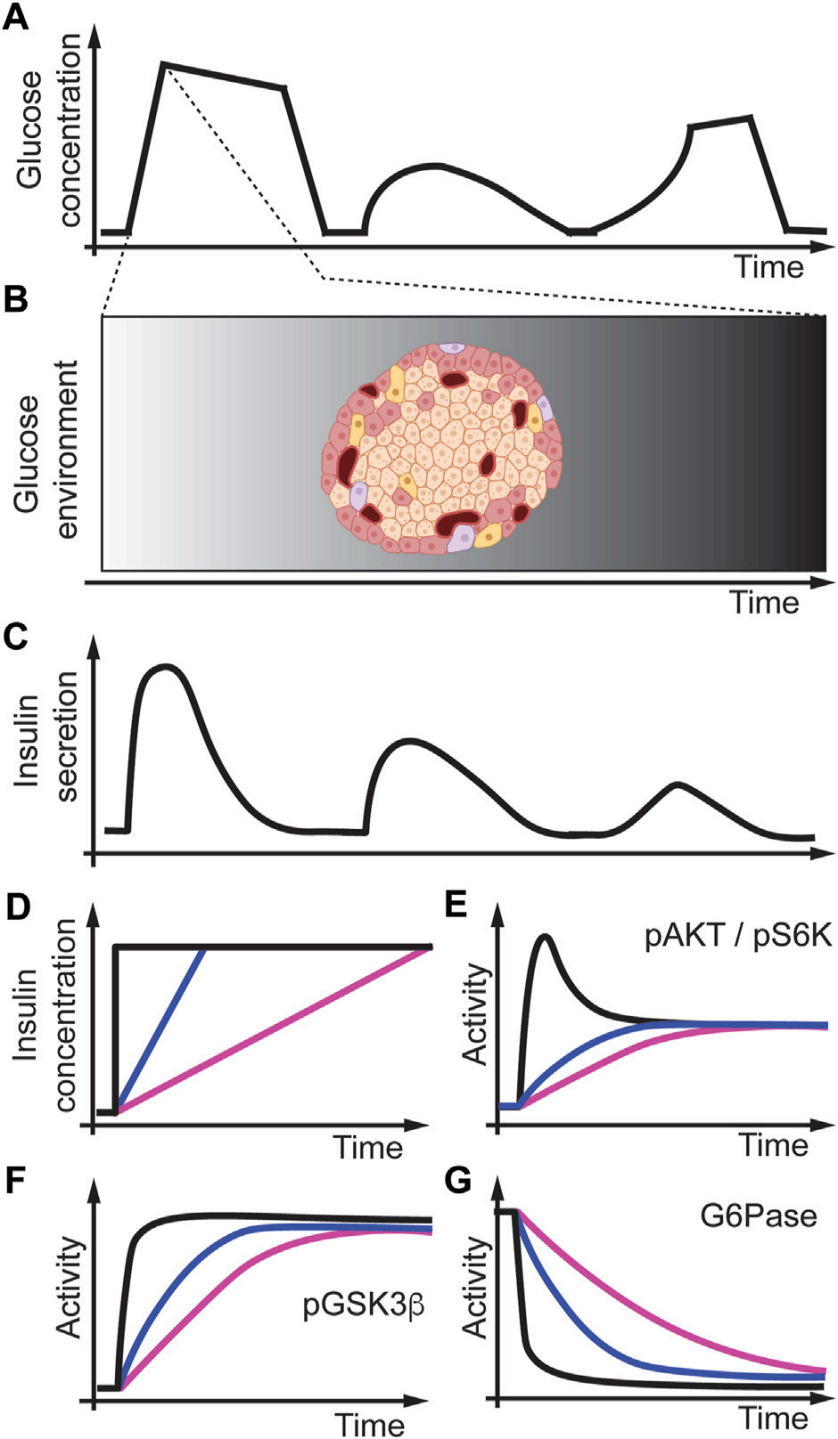
Modeling temporal dynamics of glucose and insulin in the body. **(A)** Glucose concentration measured through continuous glucose monitoring. **(B)** The islet in the pancreas may experience these glucose dynamics over time. **(C)** Different glucose concentrations trigger insulin release from the islets resulting in different temporal insulin concentration profiles. **(D)** Modeling insulin concentration changes over time *in vitro* as acute (black), fast (blue), or slow (magenta) insulin release. **(E–G)** Insulin profiles differentially regulate signaling dynamics that are different in a variety of pathways. Adapted from (Kubota et al., 2012; 2018).

A well-known example in physiology in which the concentration changes over time is the relationship between glucose uptake and insulin secretion in the body (Polonsky et al., 1988; Fernandez and Torres-Alemán, 2012). The dynamic changes in insulin secretion depend on glucose levels in the bloodstream. After a meal, glucose changes in the bloodstream in different temporal patterns as measured by continuous glucose concentration monitoring (Figure 9A). The insulin-secreting beta-cells then detect these changes in blood glucose levels in the islets of the Langerhans in the pancreas (Figure 9B). Insulin secretion from the pancreas occurs in a pulsatile manner (Figure 9C), and this is critical to maintaining insulin receptor signaling/sensitivity (Matveyenko et al., 2012; Satin et al., 2015). Insulin secretion from the islet into the portal vein allows for higher concentrations of insulin that the liver is exposed to versus other peripheral tissues. Thus, with this high insulin concentration, the liver can undergo insulin receptor desensitization if the kinetics of insulin secretion are inappropriate. To better understand how different insulin gradients regulate cell signaling, Kubota et al. (2012) used an *in vitro* system and studied Fao rat hepatoma cells, a rat liver cell line, and exposed these cells to different acute and gradual concentrations of insulin (Figure 9D) (Noguchi et al., 2013). They then measured under these conditions, activation of the Akt pathway (Figure 9E) and downstream protein phosphorylation of glycogen synthase kinase-3b (GSK3b) (Figure 9F), gluconeogenesis through glucose-6-phosphatase (G6Pase) (Figure 9G), and phosphorylation of ribosomal protein S6 kinase (pS6) (Figure 9E) as a marker of protein synthesis. Kubota et al. (2018) then found that upon acutely increasing the concentration of insulin, the activity of pAKT, pGSK3b, and pS6K signaling proteins rapidly increases. In contrast, G6Pase activity rapidly decreases (Figures 9D–G, black lines). However, when they applied gradients of different rates of insulin to the same final concentrations, they found that pAKT, pGSK3b, and pS6K increased slower, whereas G6Pase decreased slower, proportional to the rate of insulin increase (Figures 9D–G, colored lines). These results indicate that pAKT, pGSK3b, pS6K, and G6Pase are all rate sensitive and may encode a rate threshold. They also infused rats with acute or gradual increases in insulin through the mesenteric vein instead of into the portal vein and directly onto the liver (as it would be during islet insulin secretion). They found that in primary hepatocytes, pAKT, pGSK3b, and pS6K signaling is very similar to rat liver cell culture experiments indicating that in rats, these pathways are also rate-sensitive *in vivo* (Kubota et al., 2018). Interestingly, pS6K was only slightly activated in slow insulin rate conditions indicating that proliferation might be rate threshold sensitive. Further studies are needed to understand better the rate threshold mechanism in cell culture and animals.

## 9 Temporal dynamics of stressors in the kidney

**FIGURE 10:**
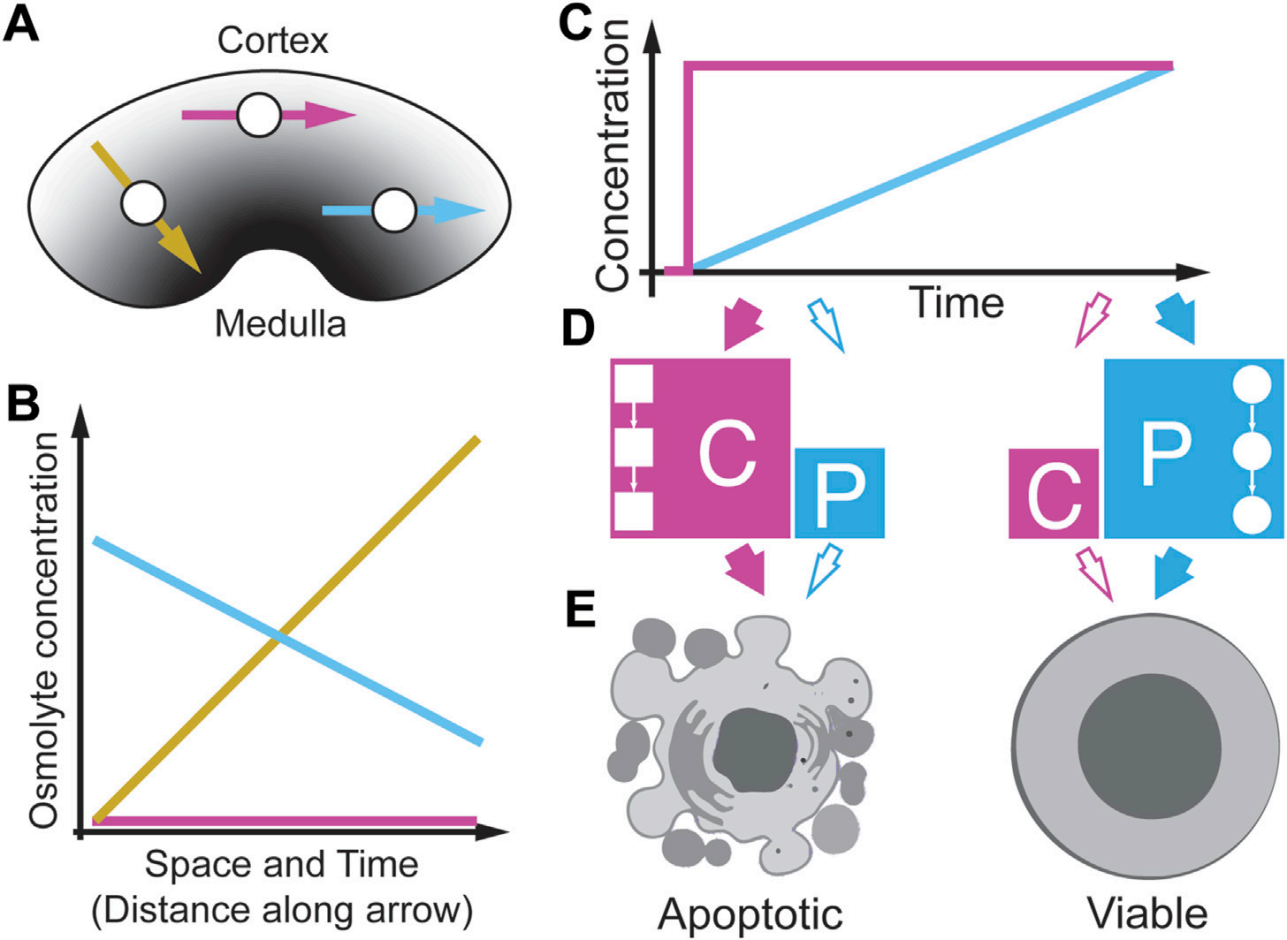
Spatio-temporal osmolyte gradients in the kidney. **(A)** Different sections of the kidney have different osmolyte concentrations from low (Cortex) to high (Medulla). Cells (white circles) can migrate in the kidney in different directions (arrows). **(B)** Cells at different positions in the kidney experience different osmolyte gradients along the direction of migration. **(C)** *In-vitro* modeling of different osmolyte concentration gradients. **(D)** Acute osmolyte changes activate caspase signaling (C) whereas gradual increase over 10 h does not activate caspase signaling but instead induces proline import (P) from the media. **(E)** Acute stress results in apoptosis (left), whereas gradual stress leads to prolin import to protect cells resulting in increases viability (right). Detailed studies indicated a rate threshold that regulate a cell fate switch from high to lower apoptosis. Adapted from (Thiemicke and Neuert, 2021).

The kidney harbors one of the most extreme environments in the human body (Figure 10A) (Neuhofer and Beck, 2005; Carlström et al., 2015; Silverthorn, 2019). Of these, the inner zone is the papillary tip which regularly has the highest osmolarity levels (Figure 10A, a dark grey area) compared to the outer zone, named the renal medulla, which has an osmolarity level similar to plasma (Figure 10A, a light grey area). Depending on the conditions, osmolarities can at least reach 1,200 mOsmol/l in humans, four times the level found in plasma (Dantzler et al., 2014; Silverthorn, 2019). How kidney cells can survive these extreme conditions is poorly understood (Neuhofer and Beck, 2005). Interestingly, the osmolyte concentrations in the kidney change dynamically over time and follow circadian rhythms (Firsov and Bonny, 2018). If these rhythms change, they can cause several kidney pathologies, indicating that the temporal patterns of osmolyte changes may be relevant for cell survival. Surprisingly, the same cell types that can function appropriately *in vivo* die in culture when exposed to acute increases in the same osmolarity (Zhang et al., 2002; Zhang et al., 2022; Sanz et al., 2008; Thiemicke and Neuert, 2021). Further studies found that a slow increase of osmolarity drastically improves cell survival of a human kidney cell line compared to a step increase (Cai et al., 2002). A follow-up study identified gene expression differences in several osmoprotective genes as the reason for the improved survival during the gradual increase (Cai et al., 2004). They suggest that kidney cells are well-adapted to extreme hyperosmolarity. However, recent studies create an image of great cellular diversity in the kidney and show that the inner medulla and the papillary tip tissue contain immune cells (Stewart et al., 2019), indicating that immune cells in the kidney need to survive strong osmolyte gradients. An open question is how these immune cells survive in this environment (Müller et al., 2019; Wilck et al., 2019). In Figure 10A, we depict three T cells that migrate through the kidney in different directions (Figure 10A, yellow, magenta, and cyan arrow), each experiencing different osmolyte gradients in space and time (Figure 10B). Interestingly, in pathological conditions, elevated levels of immune cells suggest that these cells survive in the kidney in this particular environment in various disease contexts (Sanz et al., 2008; Müller et al., 2019; Wilck et al., 2019). To better understand how immune cells survive in such harsh conditions, Thiemicke et al. (2019) modeled *in vitro* immune cell exposure to different osmolyte gradients (Figure 10C) and quantified cell viability as a phenotypic metric (Thiemicke and Neuert, 2021). They cultured cells in growth media and changed osmolarity by either acute or gradually increasing physiological NaCl concentrations. In T cells (Jurkat cells) and monocytes (THP1 cells), they observed that acute exposure to physiological NaCl concentrations in the kidney resulted in 85% cell death. In comparison, exposing the same cells to the same stressor and the same final concentration but at a linearly increasing concentration resulted in only 60% cell death, indicating a rate threshold in the cell viability. These results are consistent with observations in kidney cells from the Burg laboratory (Cai et al., 2002; Cai et al., 2004) and in several colon cancer cell lines (Zhang et al., 2022) confirming that elevated hypertonicity by NaCl can cause cell death. Thiemicke et al. then performed a temporal functional screen for 27 well-established markers of caspase signaling, stress signaling, growth, inflammation, and DNA damage to identify the principal mediators of cell death. Within the markers of caspase and stress signaling, they found differential regulation of several caspases and p38 between acute and slowly increasing stress. Upon further investigation, they found that p38 signaling does not significantly contribute to the cell death phenotype. Instead, they found caspase-mediated apoptosis as the primary contributor to cell death (Figures 10D, E). Thiemicke et al. then showed through quantitative mass spectroscopy that cell internal proline increases strongly upon hypertonic stress and that proline increases to higher levels after gradual increases of hypertonic stress (Figure 10D). Supplementing external proline to Jurkat cells protects them against acutely changing stress conditions to a similar extent but synergistically when compared to caspase inhibition, demonstrating that proline functions as a molecular osmolyte similar to betaine in the kidney (Figure 10D) (Garcia-Perez and Burg, 1991; Cai et al., 2002; Burg and Ferraris, 2008). Thiemicke et al. identified differential regulation of caspase-mediated apoptosis in acute versus gradually increasing osmolyte concentration (Figure 10E). These studies suggest that proline may be used by human cells as an osmoprotective molecule, previously only described in non-human cells. The wider implications are that gradually increasing environmental changes differentially regulate human cell fate and signaling. In addition, a better understanding of how metabolic networks are coordinated with signaling networks to control cell fate may result in a novel therapeutic avenue of intervention in pathophysiological conditions.

## 10 Summary and future directions

**FIGURE 11:**
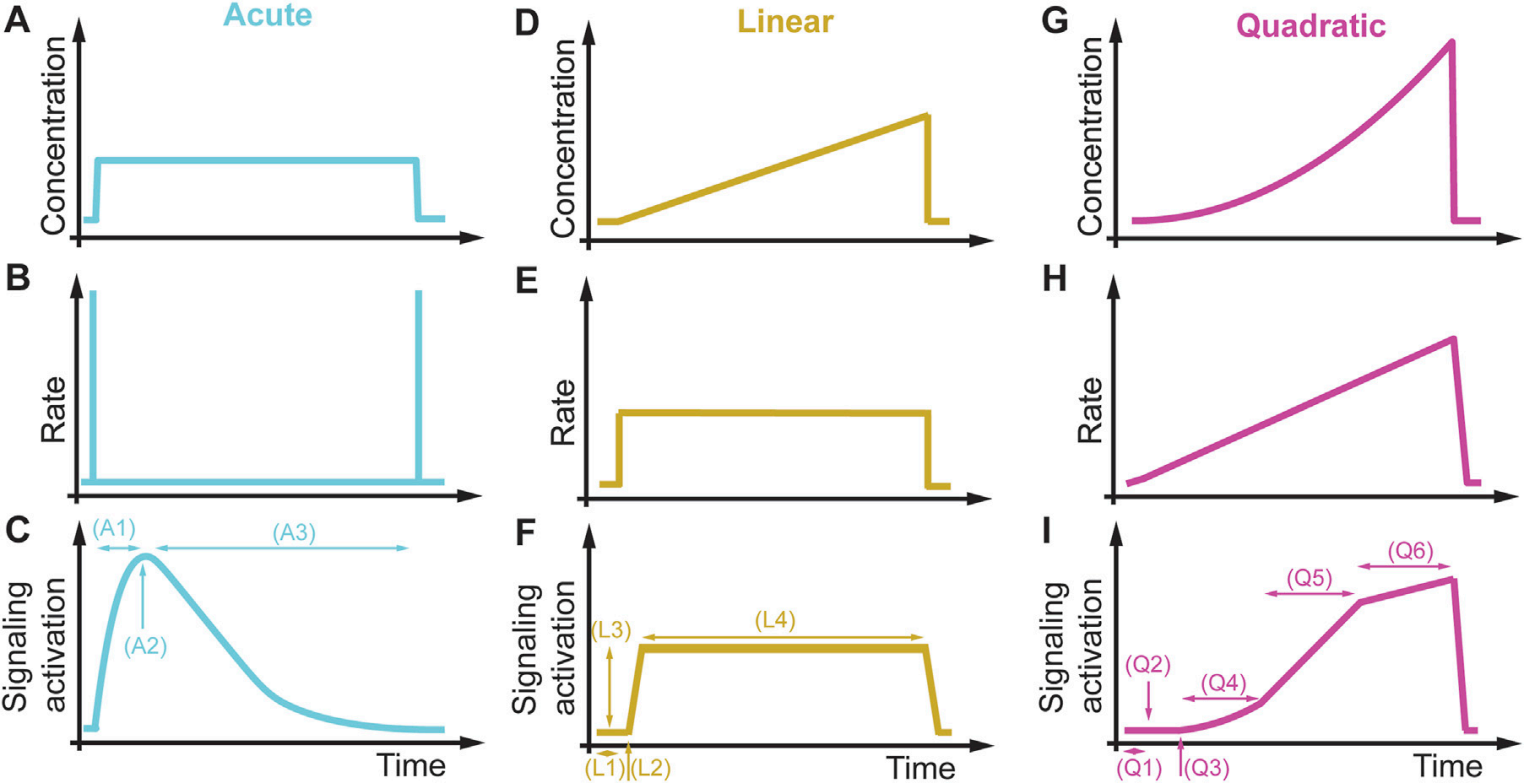
Signaling features and phenotypes in different dynamic environments. **(A–C)** Acute concentration changes **(A)** do not change the rate **(B)** and rapidly active signaling **(C)**. For an adaptive pathway, acute environmental changes probe the response time of the pathway (A1), the maximum signaling response (A2), and the adaptation time (A3). **(D–F)** A linear concentration change **(D)** results in a constant rate **(E)**, leading to prolonged signaling of an adaptive system **(F)**. From this signaling response, we can extract the delay in signaling activation (L1), the concentration activation threshold (L2), rate-dependent signaling response amplitude (L3), and the duration and type of adaptation (L4). **(G–I)** Using a quadratic concentration change **(G)**, resulting in an increasing linear rate **(H)** and a complex and non-linear signaling response **(I)**. This signaling response contains information about delay in signaling activation (Q1), concentration activation threshold (Q2), rate activation threshold (Q3), linear concentration dependence (Q4), linear rate dependence (Q5), and concentration and rate saturation threshold (Q6).

The previous sections described rate dependencies of different essential signaling pathways in other organisms and suggested that these pathways may encode a rate threshold mechanism. Additional experiments must be performed at different linear or quadratic rates to test these pathways’ proposed rate threshold mechanisms. From these experiments, one can learn if a pathway encodes a concentration, a rate, or both thresholds. The next step is identifying the protein(s) encoding the rate threshold. After this protein(s) has been placed, one might investigate the rate threshold mechanism through protein domain deletions, chemical inhibition, or overexpression studies. After identifying a rate threshold protein, a phenotypic assay will establish the biological relevance in different dynamic environments and rate threshold regulator mutants (see Figure 3) (Johnson et al., 2021). Besides rate thresholds, dynamic environments may also reveal other signaling features and associated phenotypes that cannot be observed in acute conditions but are relevant in non-acute physiological conditions. In Figure 11, we compare the pathway response to an acute, a linear, and a quadratic concentration change. The acute induction (Figures 11A–C) leads to a quick increase in signaling (A1), a maximum signaling response (A2), and then a slowly decaying and adapting signaling back to the initial signaling condition (A3). In a linear increasing condition (Figures 11D–F), we can quantify the delay in signaling activation (L1), a concentration threshold (L2), a rate-dependent signaling amplitude (L3), and the duration and type of adaptation (L4).

In comparison, the same cells and pathways exposed to a quadratic increasing condition (Figures 11G–I) lead to a delay in signaling activation (Q1), a detection of a concentration activation (Q2) and rate (Q3) activation thresholds, a linear concentration (Q4) and a linear rate dependency (Q5), and a saturation concentration and rate threshold (Q6) after which signaling is not rate dependent anymore, and an increase in stimulus signal do not lead to signaling increase. This example illustrates that non-acute conditions might be vital to unraveling hidden signaling features not observable with current acute perturbation paradigms. Comparing these different signaling profiles and signaling features indicate that unique activation profiles probe special signaling features. Linking these signaling features to phenotypes and regulatory proteins will help us better understand the basic mechanisms of signal transduction in normal cells. This data and knowledge will provide the foundation to investigate how mutated signaling proteins in a pathophysiological condition alter these signaling profiles and provide the basis to develop targeted therapeutic approach. These signaling features might also be conserved or modulated throughout evolution because many of the signaling proteins are evolutionarily conserved. Quantitative data generated in these experiments will build the foundation for developing approaches for model inference, quantitative predictions, and computational screening of combinatorial protein regulation (Rahi et al., 2017; Jashnsaz et al., 2020; 2021). The proposed approaches of non-acute cell perturbations are also amenable for dissecting many other signaling processes, such as signaling cross talk, cell cycle regulation, protein translation, phase separation, and gene regulation, which are all dependent on specific signaling dynamics. Lastly, designing drug profiles in cell culture will fill a gap in understanding drug mechanisms that will guide the design of expensive and time-consuming pharmacodynamics and pharmacokinetics studies in animals and humans and improve the translatability of non-clinical studies. Finally, studying cell physiology besides the mentioned processes in non-acute conditions might reveal many hidden biological mechanisms currently not accessible with acute treatment conditions, expanding the observable phenotypic space underlying normal and pathophysiological conditions in humans as conceptualized in Figure 2.
